# Risk factors for atrial fibrillation after lung cancer surgery: a meta-analysis

**DOI:** 10.3389/fcvm.2026.1768794

**Published:** 2026-02-11

**Authors:** Jingyu Zhang, Siyu Liu, Jiayao Li, Yuqian Wang, Ming Ni, Cheng Jiang, Songqun Huang, Zhifu Guo

**Affiliations:** 1 Department of Cardiology, Changhai Hospital, Naval Medical University, Shanghai, China; 2 Department of Nursing, Air Force Medical University, Xi'an, Shaanxi, China

**Keywords:** atrial fibrillation, lung cancer, meta-analysis, risk factors, surgery

## Abstract

**Background:**

This study aims to explore potential risk factors for atrial fibrillation (AF) following lung cancer surgery through a meta-analysis.

**Methods:**

PubMed, Embase, and the Cochrane Library databases were searched to identify all relevant studies on postoperative AF following lung cancer surgery. Inclusion criteria specified adult patients with lung cancer surgery who had clearly reported risk factors for AF. The search was conducted up to October 20, 2025. The quality of included studies was assessed using the standardized NOS scoring tool, and statistical analysis was performed using Stata 15. Data from all included studies were analyzed using a random-effects model.

**Results:**

A total of 13 articles involving 20,701 lung cancer patients were included. The meta-analysis results suggest that age >65[OR = 1.68, 95% CI (1.30, 2.16)], Postoperative high BNP [OR = 3.82, 95% CI (1.43, 10.25)], male [OR = 1.82, 95% CI (1.35, 2.45)], smoking [OR = 1.72, 95% CI (1.35, 2.21)], hypertension [OR = 1.63, 95% CI (1.08, 2.48)], patients with TNM stage II lung cancer [OR = 2.21, 95% CI (1.22, 4.01)], transfusion [OR = 3.74, 95% CI (2.28, 6.12)] were associated with an increased risk of postoperative AF after lung cancer surgery.

**Conclusions:**

This study suggesting that factors such as age >65 years, male gender, smoking, hypertension, elevated postoperative BNP levels, TNM stage II, and perioperative blood transfusion may be associated with an increased risk of postoperative AF in lung cancer patients.

**Systematic Review Registration:**

https://www.crd.york.ac.uk/PROSPERO/view/CRD420251176315, Prospero CRD420251176315.

## Background

Lung cancer ranks among the leading causes of cancer-related deaths worldwide. According to global cancer burden data, its incidence and mortality rates remain high in both men and women, with an upward trend driven by factors such as smoking and environmental pollution (1, 2). Treatment options for lung cancer primarily include surgery, radiotherapy, chemotherapy, and targeted therapy (3). Surgical resection is considered one of the most effective curative approaches for early-stage lung cancer (4). However, while lung cancer surgery significantly improves patient survival rates, postoperative complications remain a critical factor affecting patient prognosis and quality of life (5).

Atrial fibrillation (AF), a common arrhythmia, exhibits a marked increase in incidence with advancing age. AF not only increases the risk of stroke and heart failure but is also closely associated with elevated mortality rates (6, 7). Among lung cancer patients, AF is one of the common postoperative complications. Studies indicate that the incidence of AF after lung cancer surgery is higher than in the general population and is closely related to postoperative recovery, length of hospital stay, and mortality rates (8). With continuous advancements in surgical techniques and treatment methods, the prevention and management of postoperative complications have become a research focus (9). Identifying risk factors for postoperative AF in lung cancer patients is crucial for reducing its incidence and improving patient quality of life.

The pathogenesis of AF is complex, involving multiple factors including cardiac structural and electrophysiological changes, postoperative stress responses, inflammatory reactions, and oxidative stress (10). Lung cancer surgery itself poses significant physiological challenges to patients (11). Factors such as surgical trauma, anesthesia, and hemodynamic changes during the procedure may trigger or exacerbate AF (12, 13). Particularly in patients with advanced lung cancer, severe systemic inflammatory responses and cardiovascular burden following surgery may further elevate the risk of AF.

Although existing studies (14, 15) have explored the mechanisms and risk factors for postoperative AF in lung cancer patients, conclusions remain inconsistent due to limitations in sample size, study design, and regional variations. Therefore, this meta-analysis aims to comprehensively identify these risk factors, thereby informing improved postoperative management and care strategies for lung cancer patients. This approach seeks to enhance patient quality of life, reduce postoperative complications, and advance research in the prevention and treatment of cardiovascular complications following lung cancer surgery.

## Methods

This systematic evaluation and meta-analysis will strictly follow the PRISMA (Preferred Reporting Items for Systematic Reviews and Meta-Analyses) guidelines (16). And it is registered in Prospero with registration number CRD420251176315.

## Literature retrieval

This study searched PubMed, Embase, Cochrane Library, and Web of Science databases. The search cutoff date was October 20, 2025. Search terms (Mesh and free text) included: (Lung Neoplasms [Mesh] OR Lung Neoplasms [Title/Abstract]) and (Atrial Fibrillation [Mesh] OR Atrial Fibrillation [Title/Abstract]) and (Risk Factors [Mesh] OR Factor, Risk [Title/Abstract]). We will also manually search the reference lists of included studies to ensure comprehensive inclusion of relevant research.

## Inclusion and exclusion criteria

### Inclusion criteria

1. Study Type: Prospective or retrospective observational studies (cohort studies and case control).
2. Study Population: Adult patients (age > 18) with lung cancer undergoing surgery.
3. Exposure: The exposure factor is the occurrence of postoperative AF, meaning the patient develops AF following lung cancer surgery, regardless of whether the occurrence is a single episode or recurrent.
4. Study Results: Provided or calculable effect sizes for risk factors (OR values, RR values, and their 95% confidence intervals).
5. Assessable literature quality with complete data.

### Exclusion criteria

1. Studies with duplicate publications or overlapping data.
2. Case reports, conference abstracts, reviews, commentaries, or animal studies.
3. Studies that do not clearly distinguish between AF and other arrhythmias or have methodological issues impacting data reliability.
4. Studies where full-text access is unavailable.

## Study selection

During the literature screening process, two researchers independently used EndNote 21 software to initially screen the literature obtained from the search, first through the titles and abstracts, and then to exclude literature that clearly did not meet the inclusion criteria. Subsequently, the remaining literature was reviewed by reading the full text in its entirety to further determine whether it met the inclusion and exclusion criteria. In case of disagreement between the two researchers during the screening process, it would be resolved through discussion and negotiation; if the negotiation still failed to reach a consensus, a third researcher would be invited to adjudicate to ensure the objectivity and consistency of the screening process.

## Data extractions

This study was conducted by two researchers who independently extracted relevant data from the eligible literature using an Excel sheet based on the inclusion criteria. The extraction included the basic information of the study (first author, year of publication, country and study design), the basic characteristics of the study population (sample size, number of AF, gender, mean age (years) and the regression analysis. In the process of data extraction, if two investigators disagreed on the data, it would be resolved through negotiation, and if no agreement could be reached, a third investigator would adjudicate to ensure the accuracy and consistency of data extraction.

## Quality evaluation

The types of studies included in this study will be assessed using different quality assessment tools: for case-control and cohort studies, the NOS (Newcastle-Ottawa Scale) (17) quality assessment tool will be used, which evaluates the intrinsic bias of the studies through three main domains (study selectivity, comparability, and assessment of outcomes), focusing on sample selection, the relationship between exposure and relationship between exposure and outcome, and control of confounders; these quality assessment tools ensure that the included studies have a high-quality evidence base.

## Statistical analysis

In this study, the random effects model was adopted due to the high degree of heterogeneity among the included studies. The risk ratio (OR) and corresponding 95% confidence interval (CI) were extracted for each study and then pooled together. To account for the variability between studies, the random effects model was chosen, which provides a more generalized estimate of the overall effect size when there is significant heterogeneity. The heterogeneity of the combined studies was assessed using the I² statistic. If the I² value was greater than 50%, it was indicative of substantial heterogeneity, and further exploration of potential sources of this heterogeneity was required. In cases of high heterogeneity, sensitivity analyses may be performed to identify any factors that could influence the overall effect sizes. To detect publication bias, a funnel plot was generated, and its asymmetry was examined. If the funnel plot showed signs of asymmetry, Egger's test was performed to assess the statistical significance of the bias. A *p*-value of <0.05 suggests the presence of publication bias, while a *p*-value of > 0.05 indicates no significant bias. If necessary, the trim-and-fill method may be applied to adjust for any potential publication bias and verify the robustness of the results. Finally, the combined effect sizes will be reported as odds ratios (ORs) with their respective 95% confidence intervals (CIs) to facilitate the interpretation of the findings and allow for statistical inference.

## Results

### Literature search results

**Figure 1:**
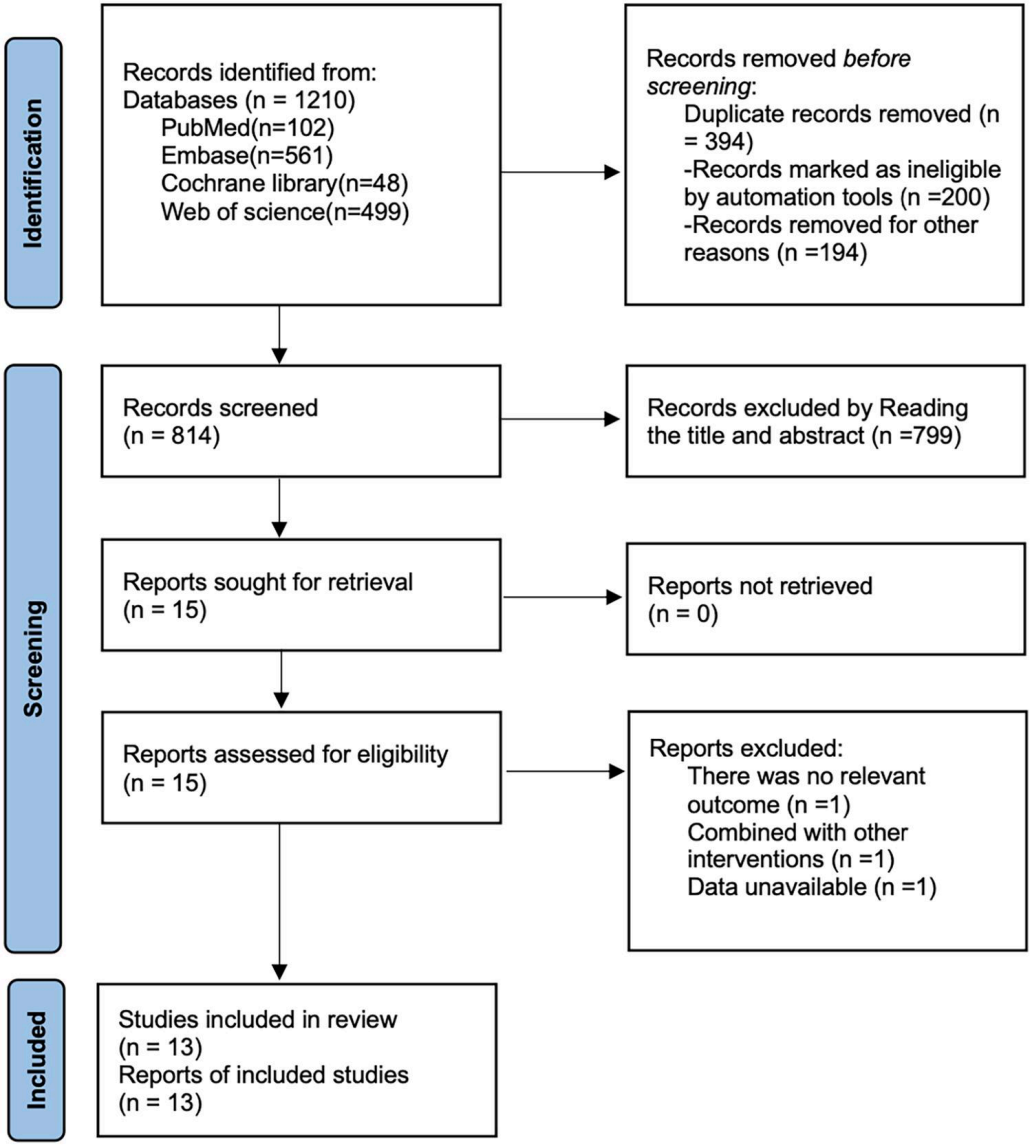
Literature search flow chart.

As shown in Figure 1, a total of 1,210 articles were retrieved from PubMed (*n* = 102), Embase (*n* = 561), Cochrane Library (*n* = 48), and Web of Science (*n* = 499). After removing 394 duplicate records, 799 articles were excluded based on title and abstract screening, and 3 articles were excluded after full-text review. Ultimately, 13 articles (18–30) were included.

### Table of basic characteristics

This study included 13 research articles: 1 case-control study and 12 cohort studies, involving a total of 20,701 lung cancer patients. Among them, 2,436 developed AFs, with ages ranging from 62.3 to 70.2 years. Detailed baseline characteristics are presented in Table 1.

**Table 1:** Table of basic characteristics.

Study	Year	Country	Study design	Sample size	No of AF	Gender (M/F)	Mean age (years)	Regression model
Cardinale	2007	Italy	cohort study	400	72	271/129	64	Multivariable logistic regression
Chen	2025	China	cohort study	208	41	124/84	69	Multivariable logistic regression
Ciszewski	2013	Poland	cohort study	117	19	92/25	68	Multivariable logistic regression
Figas	2007	Poland	cohort study	80	24	60/20	61.6	Multivariable logistic regression
Gong	2021	China	cohort study	344	55	222/122	65	Multivariable logistic regression
Gong	2025	China	cohort study	369	70	269/100	62.3	Multivariable logistic regression
Han	2024	China	cohort study	2,920	153	1,345/1,675	70.1	Multivariable logistic regression
Imperatori	2012	Italy	cohort study	454	45	369/85	65.4	Multivariable logistic regression
Ishibashi	2020	Japan	cohort study	947	49	547/400	70.2	Multivariable logistic regression
Ivanovic	2014	Canada	cohort study	363	43	168/195	70	Multivariable logistic regression
Iwata	2016	Japan	cohort study	377	38	300/77	68.3	Multivariable logistic regression
Onaitis	2010	USA	cohort study	13,906	1,755	7,906/6,000	67	Multivariable logistic regression
Zhang	2024	China	case-control	216	72	144/72	62.3	Multivariable logistic regression

### Risk of bias results

As shown in Table 2, seven studies scored 9 points, four studies scored 8 points, and two studies scored 7 points. All articles included in this study were of high quality.

**Table 2:** NOS scores results.

Cohort study									
Study	Representativeness of the exposed group	Selection of non-exposed groups	Determination of exposure factors	Identification of outcome indicators not yet to be observed at study entry	Comparability of exposed and unexposed groups considered in design and statistical analysis	design and statistical analysis	Adequacy of the study's evaluation of the outcome	Adequacy of follow-up in exposed and unexposed groups	Total scores
Cardinale 2007	*	*	*	*	**	*	*	*	9
Chen 2025	*	*	*	/	**	*	*	*	8
Ciszewski 2013	*	*	*	/	**	*	*	*	8
Figas 2007	*	*	*	*	**	*	*	*	9
Gong 2021	*	*	*	*	**	*	*	*	9
Gong 2025	*	*	*	*	**	*	*	*	9
Han 2024	*	*	*	/	**	/	*	*	7
Imperatori 2012	*	*	*	*	**	*	*	*	9
Ishibashi 2020	*	*	*	/	**	*	*	*	8
Ivanovic 2014	*	*	*	/	**	*	*	*	8
Iwata 2016	*	*	*	*	**	*	*	*	9
Onaitis 2010	*	*	*	/	**	/	*	*	7
Case control									
Study	Is the case definition adequate?	Representativeness of the cases	Determination of control group	Definition of Controls	Comparability of cases and controls based on the design or analysis	Ascertainment of exposure	Same method of ascertainment for cases and controls	Non response	Total scores
Zhang 2024	*	*	*	*	**	*	*	*	9

## Meta-analysis results

### Age > 65

**Figure 2:**
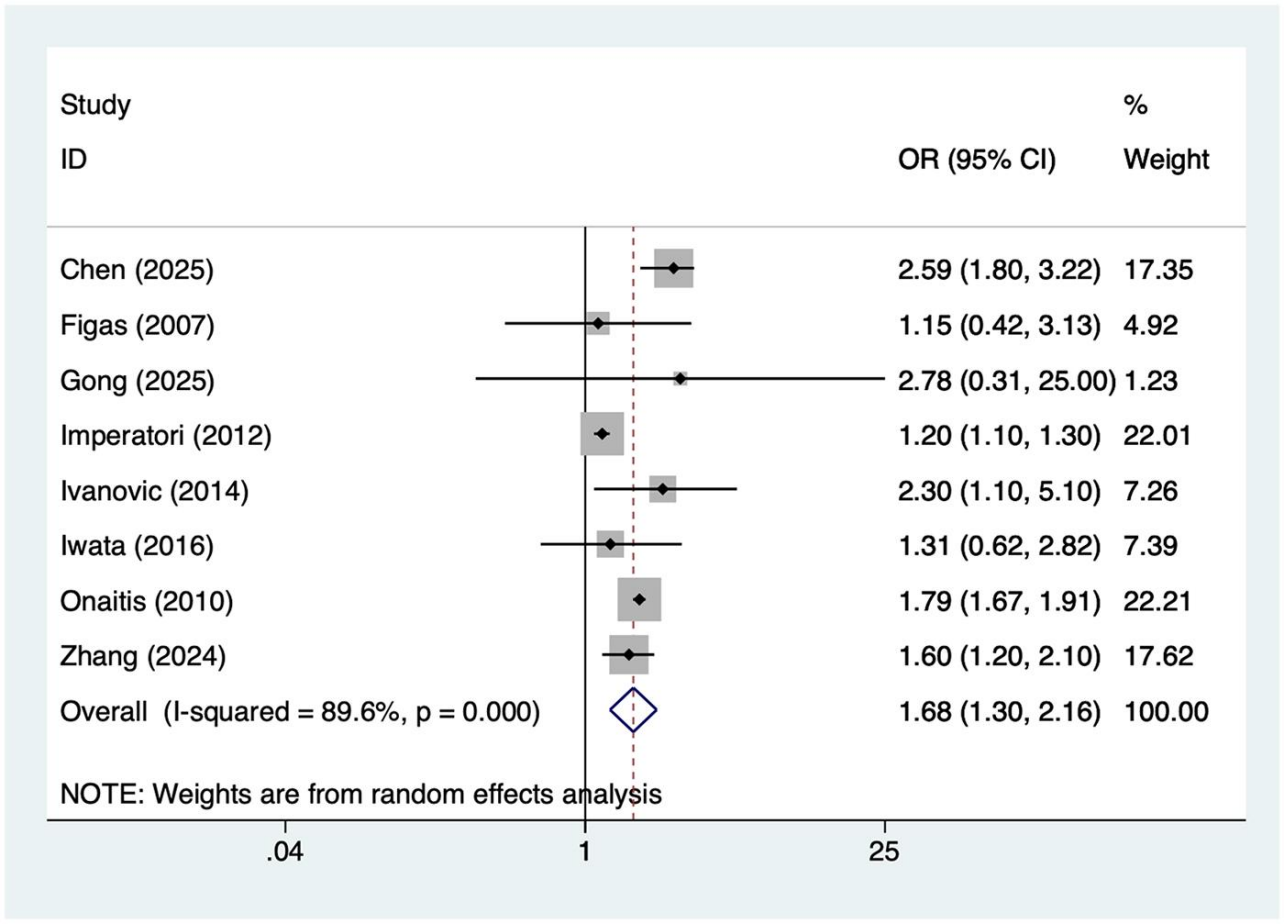
Forest plot of the meta-analysis of age >65 years.

8 studies reported age >65 years. Heterogeneity was high (I² = 89.6%, *P* = 0.001), so a random-effects model was applied. The pooled analysis (Figure 2) indicated that age >65 was associated with an increased risk of postoperative AF after lung cancer surgery [OR = 1.68, 95% CI (1.30, 2.16)]. Sensitivity analysis (Supplementary Figure S1) showed that the results were stable and not influenced by any single study.

### Postoperative high BNP

**Figure 3:**
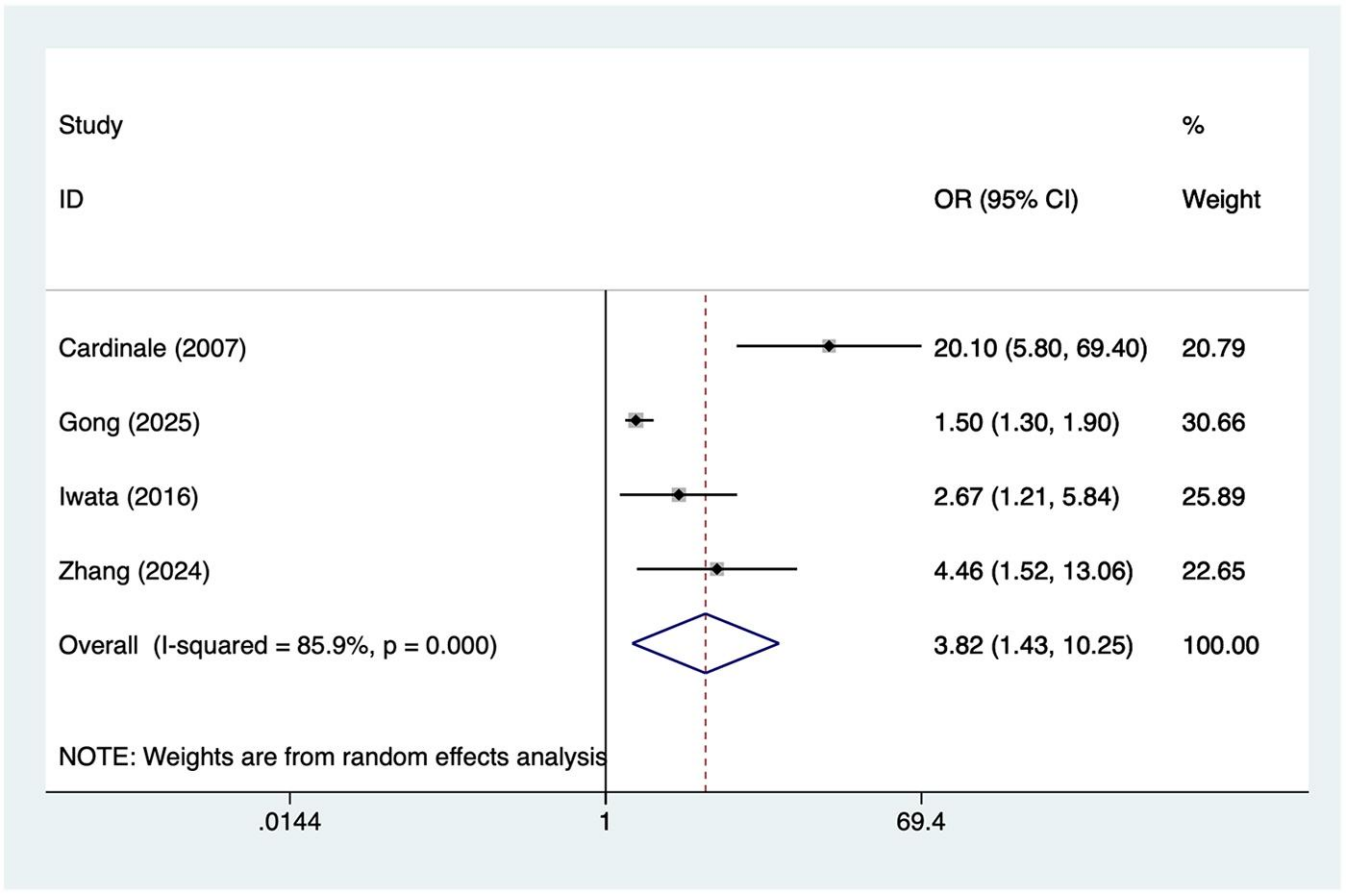
Forest plot of the meta-analysis of postoperative high BNP.

4 studies reported Postoperative high BNP. Heterogeneity was high (I² = 85.9%, *P* = 0.001), so a random-effects model was applied. The pooled analysis (Figure 3) indicated that Postoperative high BNP was associated with an increased risk of postoperative AF after lung cancer surgery [OR = 3.82, 95% CI (1.43, 10.25)]. Sensitivity analysis (Supplementary Figure S2) showed that the results were stable and not influenced by any single study.

### Male

**Figure 4:**
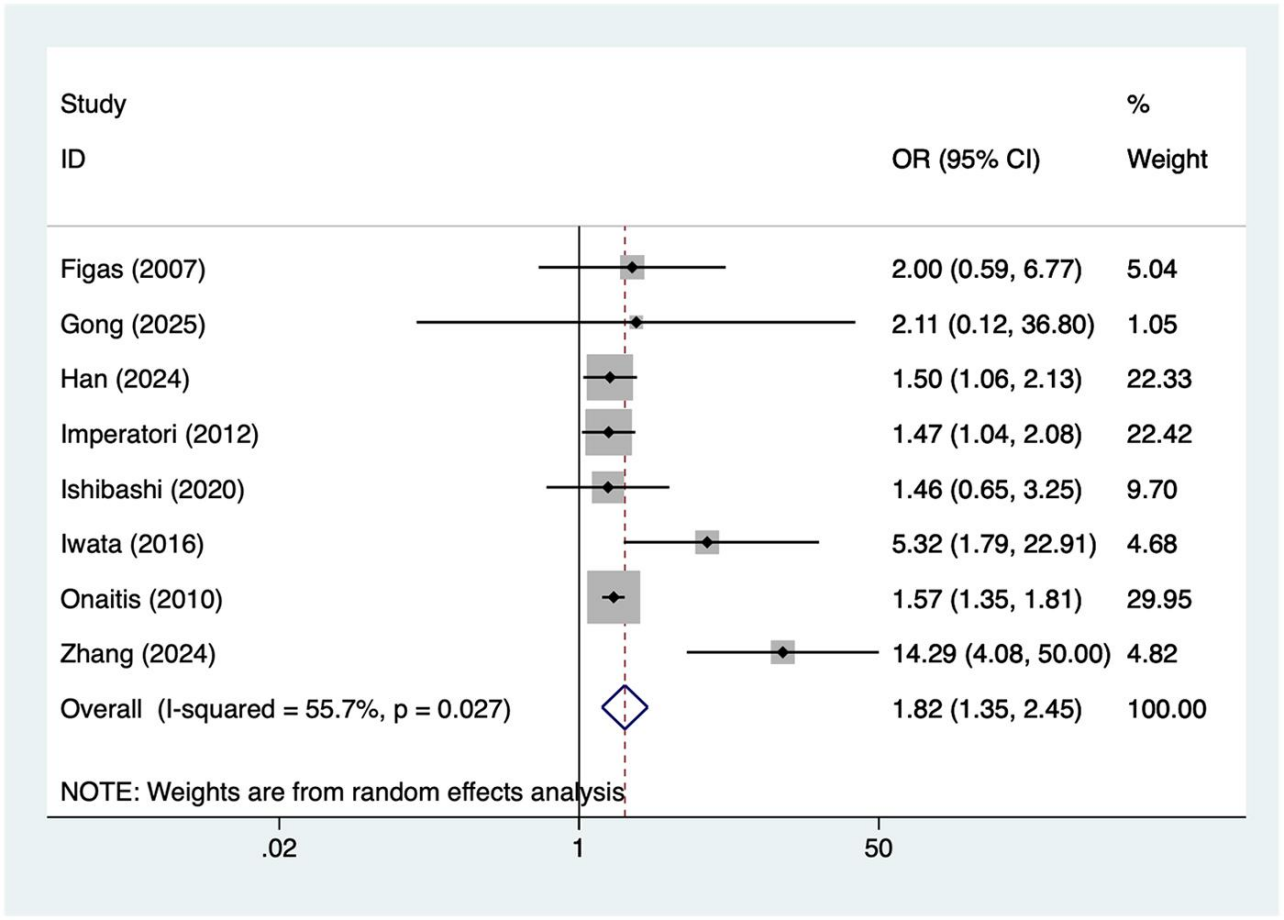
Forest plot of the meta-analysis of male.

8 studies reported male. Heterogeneity was moderate (I² = 55.9%, *P* = 0.027), so a random-effects model was applied. The pooled analysis (Figure 4) indicated that male was associated with an increased risk of postoperative AF after lung cancer surgery [OR = 1.82, 95% CI (1.35, 2.45)]. Sensitivity analysis (Supplementary Figure S3) showed that the results were stable and not influenced by any single study.

### Smoking

**Figure 5:**
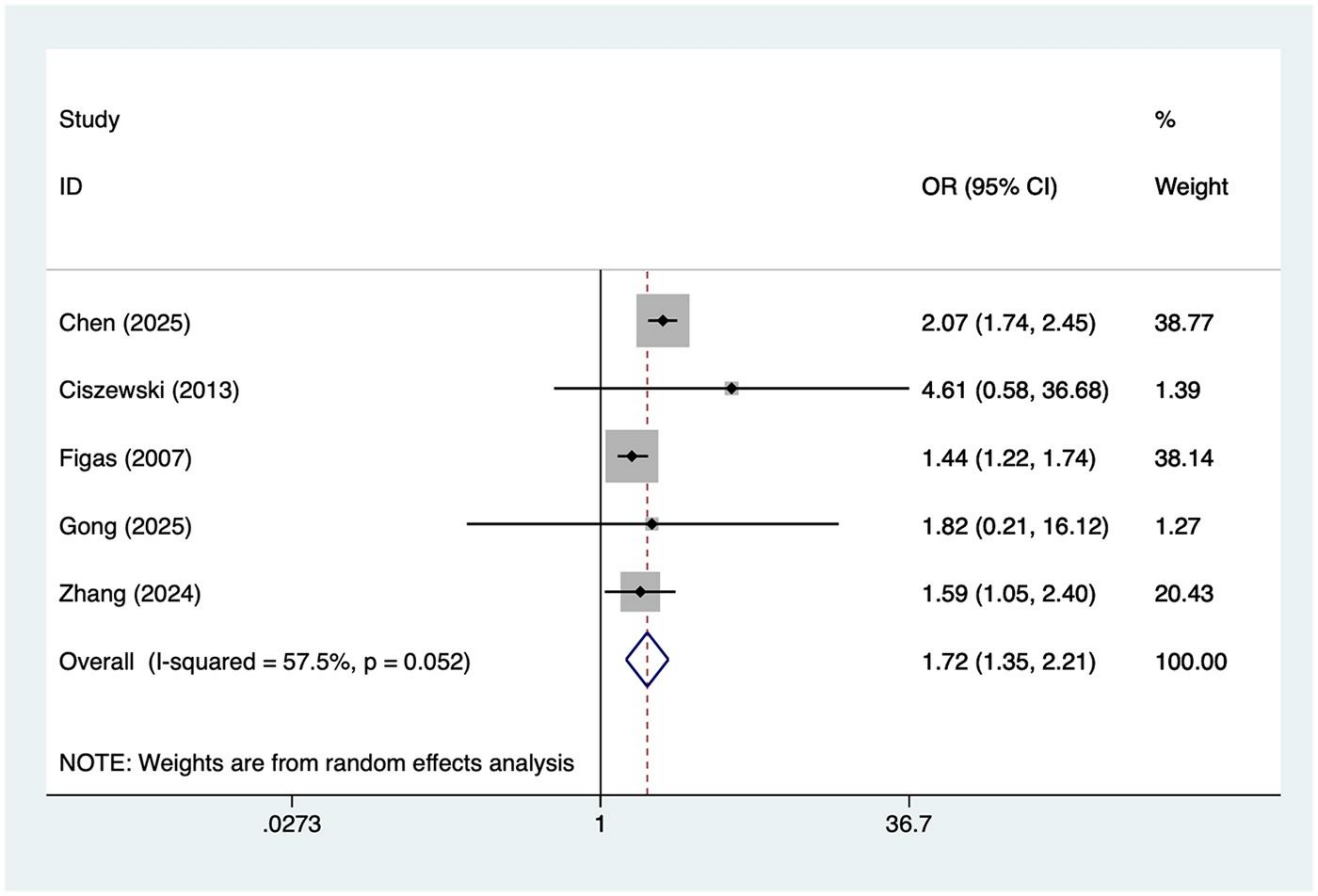
Forest plot of the meta-analysis of smoking.

5 studies reported smoking. Heterogeneity was moderate (I² = 57.5%, *P* = 0.052), so a random-effects model was applied. The pooled analysis (Figure 5) indicated that smoking was associated with an increased risk of postoperative AF after lung cancer surgery [OR = 1.72, 95% CI (1.35, 2.21)]. Sensitivity analysis (Supplementary Figure S4) showed that the results were stable and not influenced by any single study.

### Hypertension

**Figure 6:**
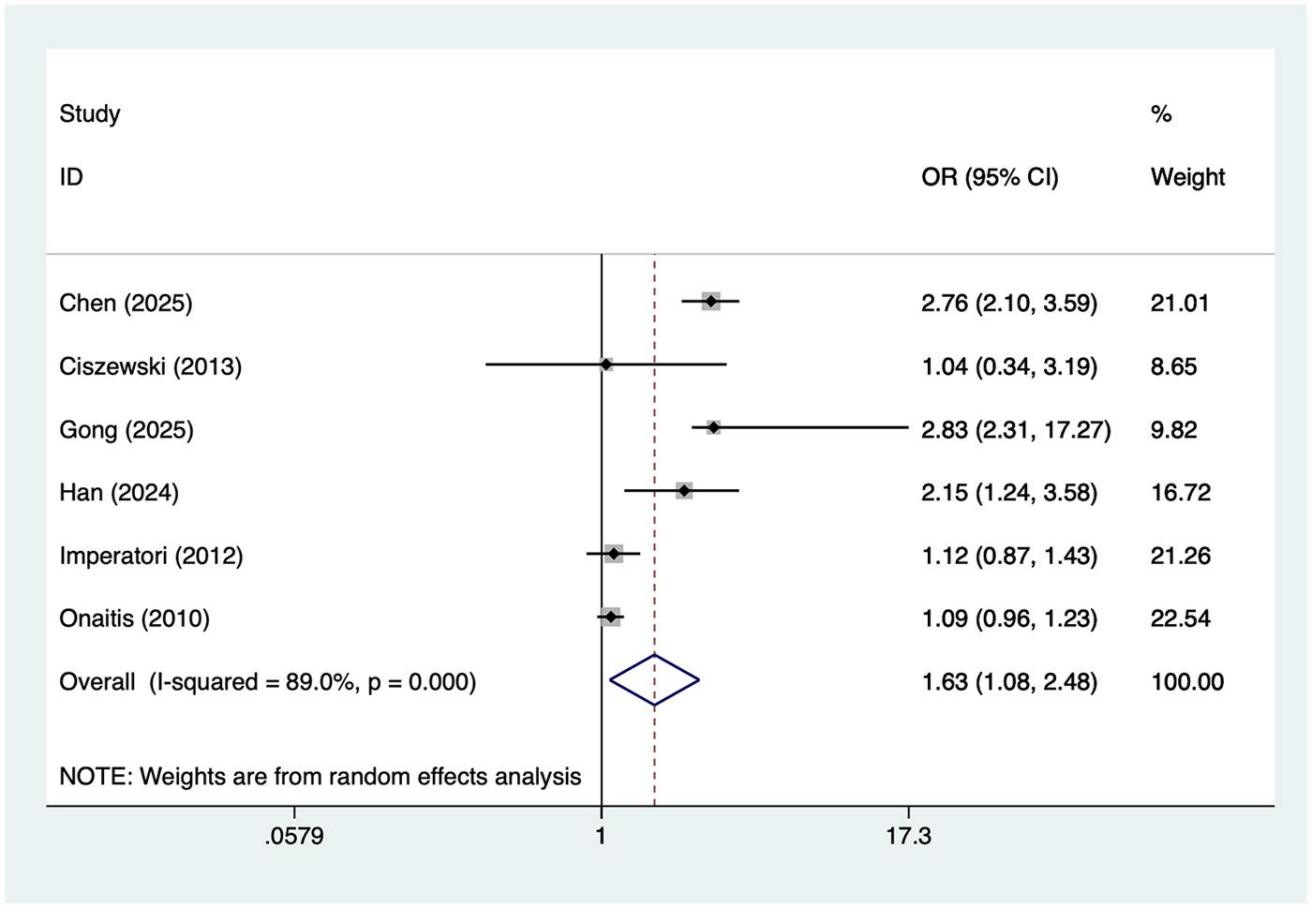
Forest plot of the meta-analysis of hypertension.

6 studies reported hypertension. Heterogeneity was high (I² = 89.0%, *P* = 0.001), so a random-effects model was applied. The pooled analysis (Figure 6) indicated that hypertension was associated with an increased risk of postoperative AF after lung cancer surgery [OR = 1.63, 95% CI (1.08, 2.48)]. Sensitivity analysis (Supplementary Figure S5) showed that the results were stable and not influenced by any single study.

### Patients with TNM stage II lung cancer

**Figure 7:**
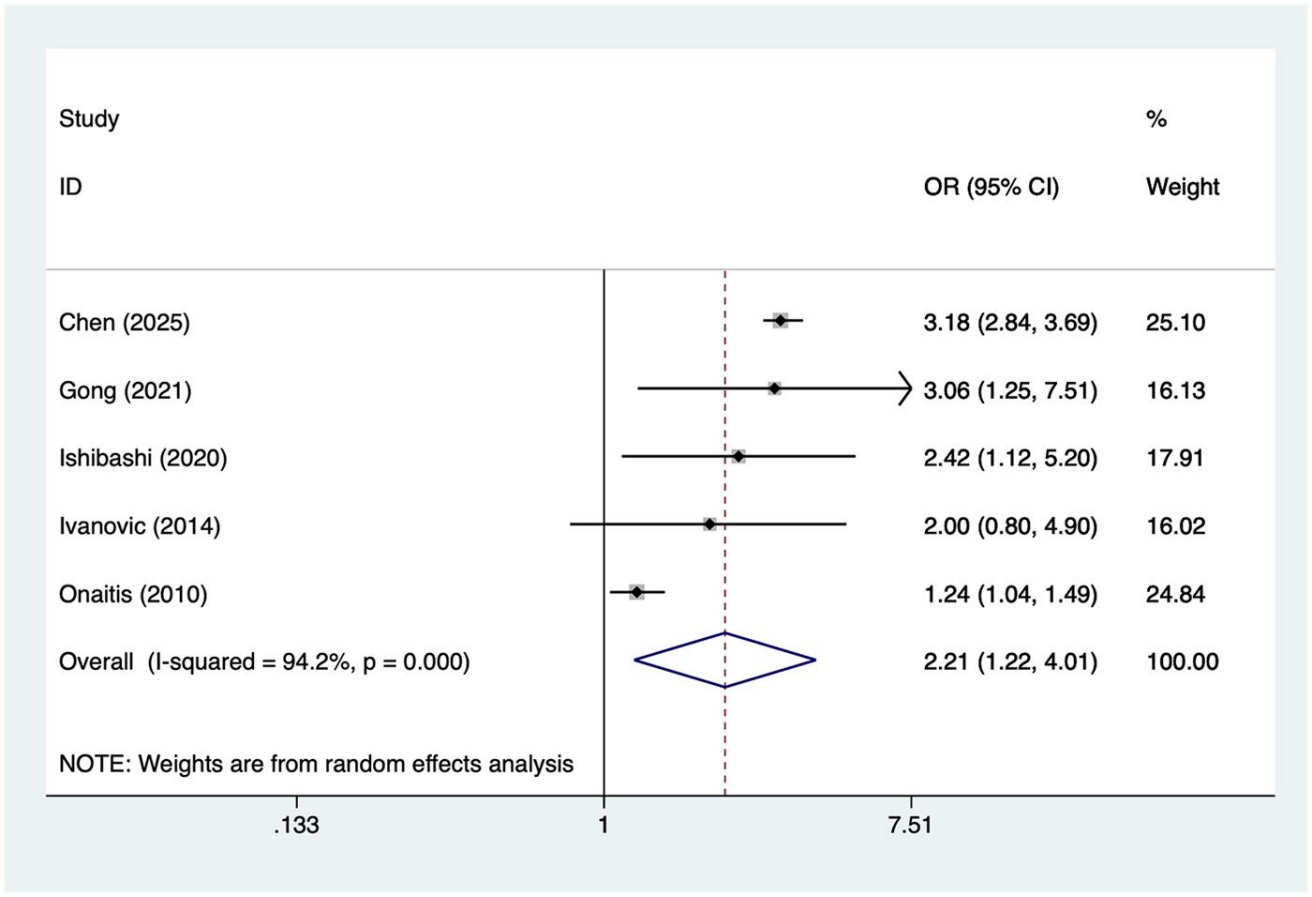
Forest plot of the meta-analysis of patients with TNM stage II lung cancer.

5 studies reported patients with TNM stage II lung cancer. Heterogeneity was high (I² = 94.2%, *P* = 0.001), so a random-effects model was applied. The pooled analysis (Figure 7) indicated that patients with TNM stage II lung cancer was associated with an increased risk of postoperative AF after lung cancer surgery [OR = 2.21, 95% CI (1.22, 4.01)]. Sensitivity analysis (Supplementary Figure S6) showed that the results were stable and not influenced by any single study.

### Transfusion

**Figure 8:**
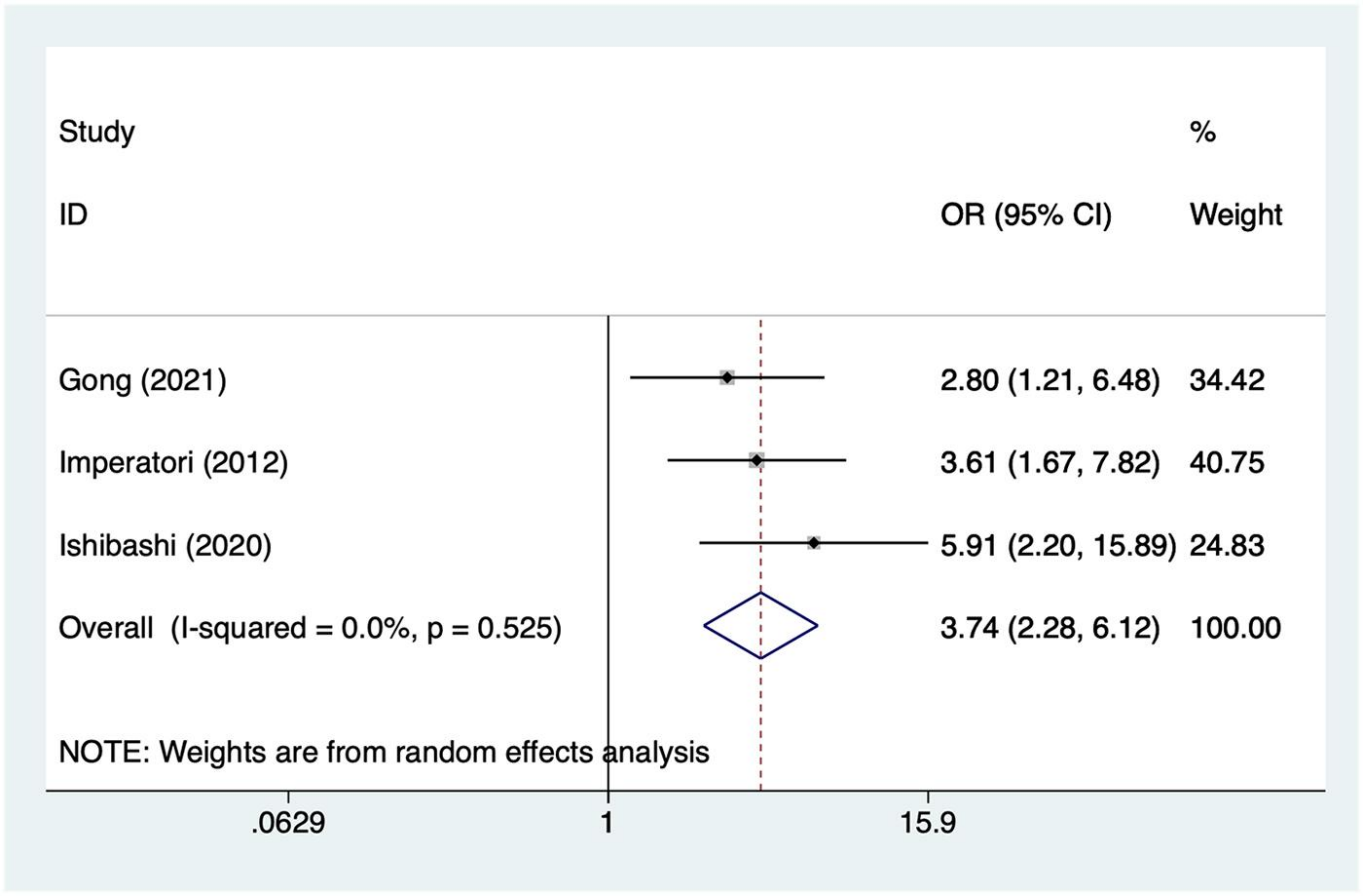
Forest plot of the meta-analysis of transfusion.

3 studies reported transfusion. Heterogeneity was low (I² = 0%, *P* = 0.525), so a random-effects model was applied. The pooled analysis (Figure 8) indicated that transfusion was associated with an increased risk of postoperative AF after lung cancer surgery [OR = 3.74, 95% CI (2.28, 6.12)].

### Publication bias

This study employed the Egger test and funnel plots to assess publication bias. Results (Supplementary Figures S7–S13) indicated that age >65 (*P* = 0.13), postoperative elevated BNP (*P* = 0.59), male gender (*P* = 0.18), smoking (*P* = 0.94), hypertension (*P* = 0.58), patients with TNM stage II lung cancer (*P* = 0.48), and transfusion (*P* = 0.09) indicated a low likelihood of publication bias. However, due to the limited number of studies on BNP and blood transfusion, readers should exercise caution when interpreting these findings.

### Meta-regression

This study employed meta-regression to explore specific sources of heterogeneity in patients with Age >65 years, Postoperative high BNP, hypertension, and TNM stage II lung cancer. Results (Supplementary Table S1) indicate that Year, Country, and study design were not sources of heterogeneity.

## Discussion

This study systematically integrated existing research evidence on risk factors associated with postoperative AF in lung cancer patients. Data from multiple studies were combined using a random-effects model, and sensitivity analyses confirmed the stability of the results. Overall, age >65 years, elevated postoperative BNP levels, male gender, smoking, hypertension, TNM stage II, and intraoperative blood transfusion were all significantly associated with an increased risk of postoperative AF in lung cancer patients.

First, age >65 years has been demonstrated to be a significant risk factor for postoperative AF in lung cancer patients. Our findings indicate that elderly patients face a significantly higher risk of postoperative AF compared to younger patients (OR = 1.68). This observation aligns with results from cardiac surgery and thoracic surgery literature. Aging is closely associated with atrial structural remodeling, increased fibrosis, shortened effective refractory period, and autonomic dysregulation, making patients more susceptible to AF under surgical stress (31). Furthermore, elderly patients often present with comorbidities such as hypertension, coronary artery disease, and pulmonary disorders, which further exacerbate atrial pressure and inflammatory responses, providing a pathological basis for arrhythmia triggering (32). Therefore, elderly lung cancer patients should be prioritized for perioperative monitoring and management. Comprehensive preoperative cardiac function assessment and optimized management of comorbidities may help reduce the incidence of AF.

Second, this study identified elevated postoperative BNP levels as one of the most significant risk factors for AF (OR = 3.82). As a sensitive indicator of increased ventricular pressure or volume load, elevated BNP suggests myocardial stretching and heightened cardiac stress. Following lung resection, reduced pulmonary tissue volume alters pulmonary circulation dynamics (33). Concurrent postoperative inflammatory responses may place the heart under elevated stress, triggering neurohumoral dysregulation and atrial electrophysiological alterations that predispose to AF (34). Previous studies have demonstrated that elevated postoperative BNP predicts multiple adverse cardiac outcomes, including AF, heart failure, and mortality. These findings further reinforce the clinical value of BNP as an early risk assessment indicator (35). Therefore, enhanced continuous ECG monitoring is recommended for patients with elevated postoperative BNP, with prophylactic drug therapy (beta-blockers or amiodarone) administered when necessary (36).

Regarding gender, this study revealed a higher risk of postoperative AF in male lung cancer patients compared to females (OR = 1.82). Evidence indicates that men are generally more prone to AF in the general population, potentially related to differences in sex hormone levels, atrial structure, and autonomic nervous system regulation. Higher smoking rates and more prevalent cardiovascular risk factors among men may also contribute to this mechanism (37). Although the specific biological mechanisms require further clarification, clinicians should recognize the higher susceptibility of male patients and enhance postoperative AF monitoring and risk assessment.

Smoking, another significant risk factor (OR = 1.72), exhibits a well-established biological basis for its association with AF. Smoking can induce arrhythmias through multiple pathways, including increased oxidative stress, exacerbated inflammatory responses, atrial structural remodeling, and autonomic imbalance. Additionally, long-term smokers often develop chronic obstructive pulmonary disease (COPD), whose lung function impairment and increased right ventricular workload further elevate the risk of AF (38). Given the high prevalence of smoking among lung cancer patients, this factor significantly influences postoperative arrhythmias (39). Preoperative smoking cessation and postoperative pulmonary function management should be prioritized clinical interventions.

Hypertension was also confirmed to be significantly associated with AF (OR = 1.63). Hypertension-induced left ventricular hypertrophy, atrial enlargement, and atrial fibrosis form a critical foundation for AF development. Surgical stress and perioperative hemodynamic fluctuations further increase cardiac burden in hypertensive patients, predisposing them to arrhythmias (40). Consistent with prior cardiothoracic surgery literature, this study underscores the importance of perioperative blood pressure management (41). Optimizing blood pressure control and avoiding significant intraoperative fluctuations or rapid changes may help reduce the risk of AF.

This study found that patients with TNM stage II had a significantly higher risk of AF (OR = 2.21) compared to other stages. This result may reflect the impact of tumor progression on patients' overall physical condition, inflammatory levels, and nutritional status. Patients with higher tumor burden often undergo more complex procedures, experience greater surgical trauma, and exhibit stronger inflammatory responses during surgery, thereby increasing the risk of arrhythmias (42). Additionally, tumor-induced metabolic alterations and immune dysregulation may contribute to AF development. Although existing literature on this association is limited, this study provides new evidence suggesting enhanced postoperative cardiac monitoring for patients with advanced lung cancer.

Among perioperative factors, blood transfusion was found to significantly increase the risk of AF (OR = 3.74). Transfusion may elevate cardiac stress through mechanisms such as triggering inflammatory responses, altering blood viscosity, and inducing volume overload. Furthermore, transfusion is often associated with complex conditions including surgical trauma, significant blood loss, and prolonged operative time—all factors potentially triggering postoperative AF (43). Reducing transfusion rates, adopting restrictive transfusion strategies, and optimizing intraoperative hemostasis techniques may all contribute to decreasing AF events.

In this study, we highlight the potential role of cardiac ultrasound imaging techniques, particularly speckle tracking echocardiography, in atrial fibrillation risk stratification. Speckle tracking echocardiography sensitively detects subclinical left atrial dysfunction and pathological remodeling even in the absence of overt left atrial enlargement, providing early risk indicators for atrial fibrillation development. These left atrial strain parameters, especially reservoir strain, have emerged as sensitive markers for assessing atrial myocardial dysfunction, aiding in the identification of patients at high preoperative risk for AF (44). Particularly in lung cancer surgery, combining traditional clinical and biochemical risk factors with these advanced imaging techniques for preoperative assessment significantly enhances the predictive capability for postoperative AF occurrence. Looking ahead, the ongoing advancement of cardiac imaging technologies—particularly the application of speckle tracking echocardiography—will provide crucial complementary tools for personalized preoperative risk assessment and management (45). This will facilitate the early detection of potential arrhythmia risks and guide the development of tailored treatment strategies.

## Strengths and limitations

This study possesses multiple strengths. First, it was conducted in strict accordance with the methodological standards for systematic reviews and meta-analyses. The search scope covered multiple Chinese and English databases, minimizing literature omission and enhancing the comprehensiveness and reliability of the research. Second, by extracting multiple potential risk factors associated with postoperative AF in lung cancer patients, this study systematically evaluated multidimensional factors including preoperative characteristics, intraoperative procedures, and postoperative physiological changes, thereby enhancing the clinical interpretability of the results. Third, the random-effects model was uniformly applied for meta-analysis, adequately accounting for the impact of study heterogeneity on overall effects and ensuring robust results. Furthermore, sensitivity analyses were conducted to test the robustness of all significant factors, revealing no substantial influence from individual studies and further supporting the reliability of our conclusions.

However, several limitations should be noted in this study. First, most of the included studies were observational, which makes it challenging to eliminate selection bias, information bias, and other potential confounders. These factors may have impacted the strength of our causal inferences. Second, there were discrepancies in the definitions of risk factors, diagnostic criteria, and AF monitoring methods across studies, which could have introduced heterogeneity. Moreover, for certain risk factors, such as blood transfusion and postoperative BNP levels, only a limited number of studies (3, 4) were available. As such, the stability of these findings needs further confirmation through additional high-quality research. Finally, several studies lacked a standardized follow-up period or did not specify the time window during which AF occurred, which could have influenced the estimation of the true postoperative AF incidence rate. These factors, along with the high heterogeneity and the limited number of studies for certain variables, reduce the robustness of our results.

## Conclusion

This study suggesting that factors such as age >65 years, male gender, smoking, hypertension, elevated postoperative BNP levels, TNM stage II, and perioperative blood transfusion may be associated with an increased risk of postoperative AF in lung cancer patients. These factors involve patient baseline conditions, tumor characteristics, and perioperative physiological stress, potentially playing a role in the pathogenesis of AF. Although this study provides valuable reference information, its conclusions require further validation in more high-quality prospective studies due to limitations in the design types and heterogeneity of the included studies. Overall, identifying potential high-risk factors can assist clinicians in enhancing perioperative monitoring and risk management, thereby providing a basis for improving postoperative arrhythmia-related outcomes in lung cancer patients.

## Data Availability

The original contributions presented in the study are included in the article/Supplementary Material, further inquiries can be directed to the corresponding authors.
