# Supplementary material for: Risk factors for atrial fibrillation after lung cancer surgery: a meta-analysis
Source: Front Cardiovasc Med. 2026 Feb 11;13:1768794. doi: 10.3389/fcvm.2026.1768794 (PMC12933428; doi:10.3389/fcvm.2026.1768794)
Supplement: Supplementary file 1 [file Datasheet1.docx]

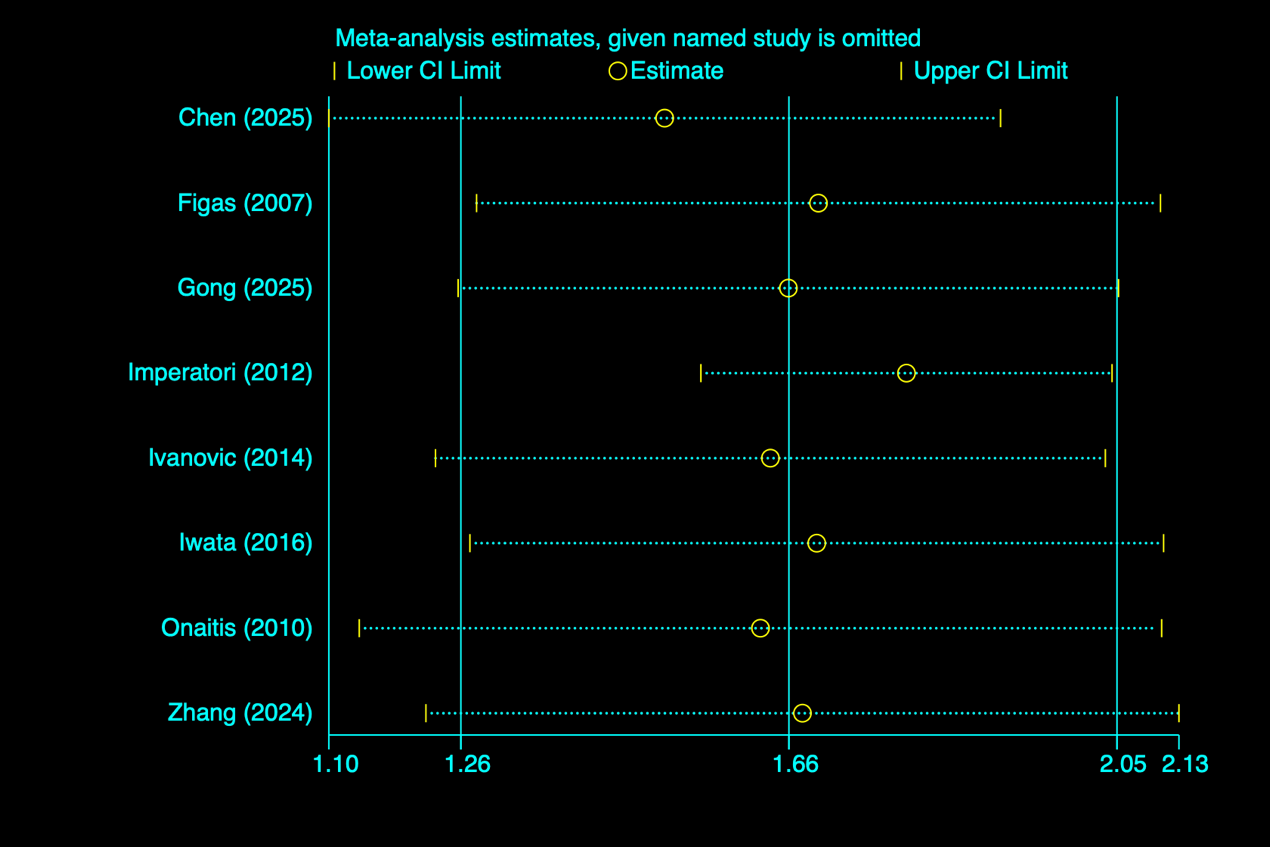


Figure S1 Sensitivity analysis for age >65 years


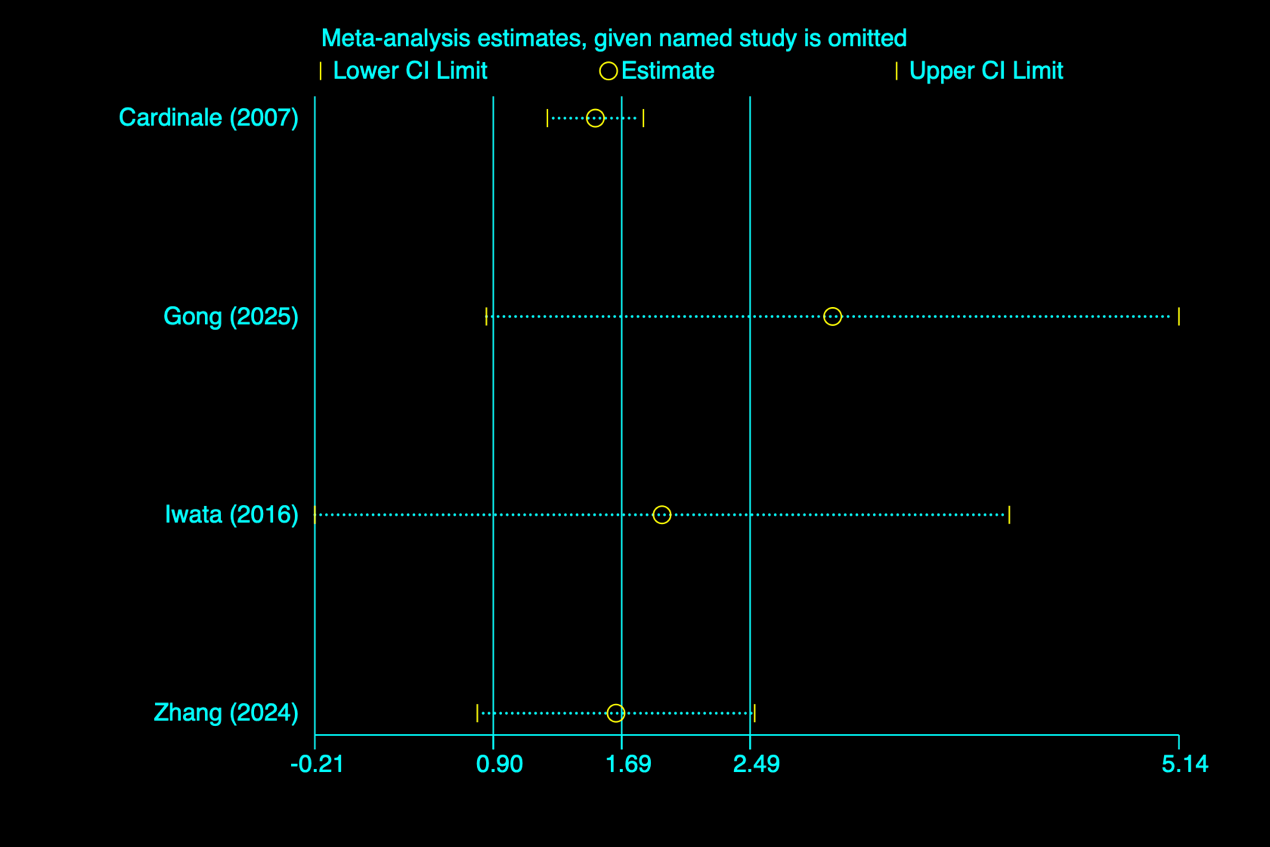


Figure S2 Sensitivity analysis for Postoperative high BNP


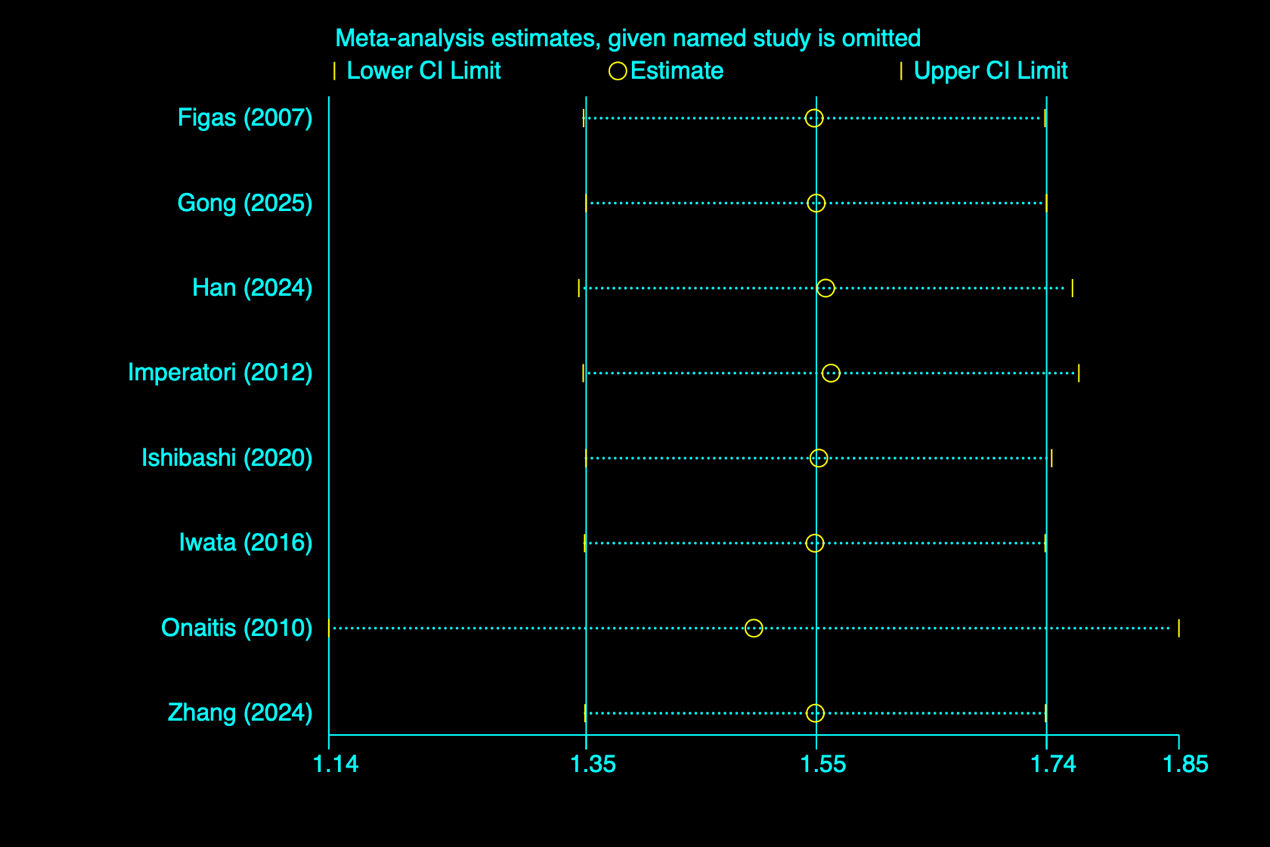


Figure S3 Sensitivity analysis for male


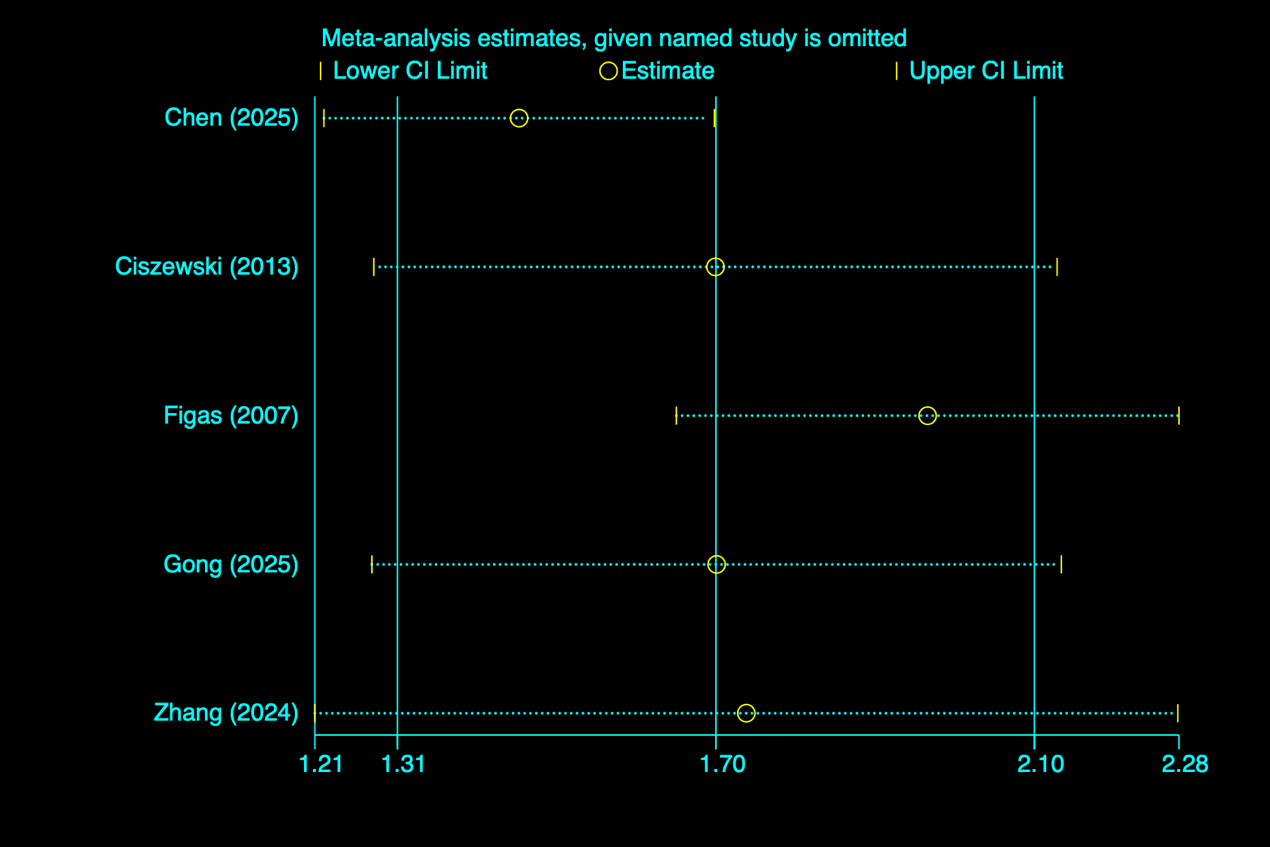


Figure S4 Sensitivity analysis for smoking


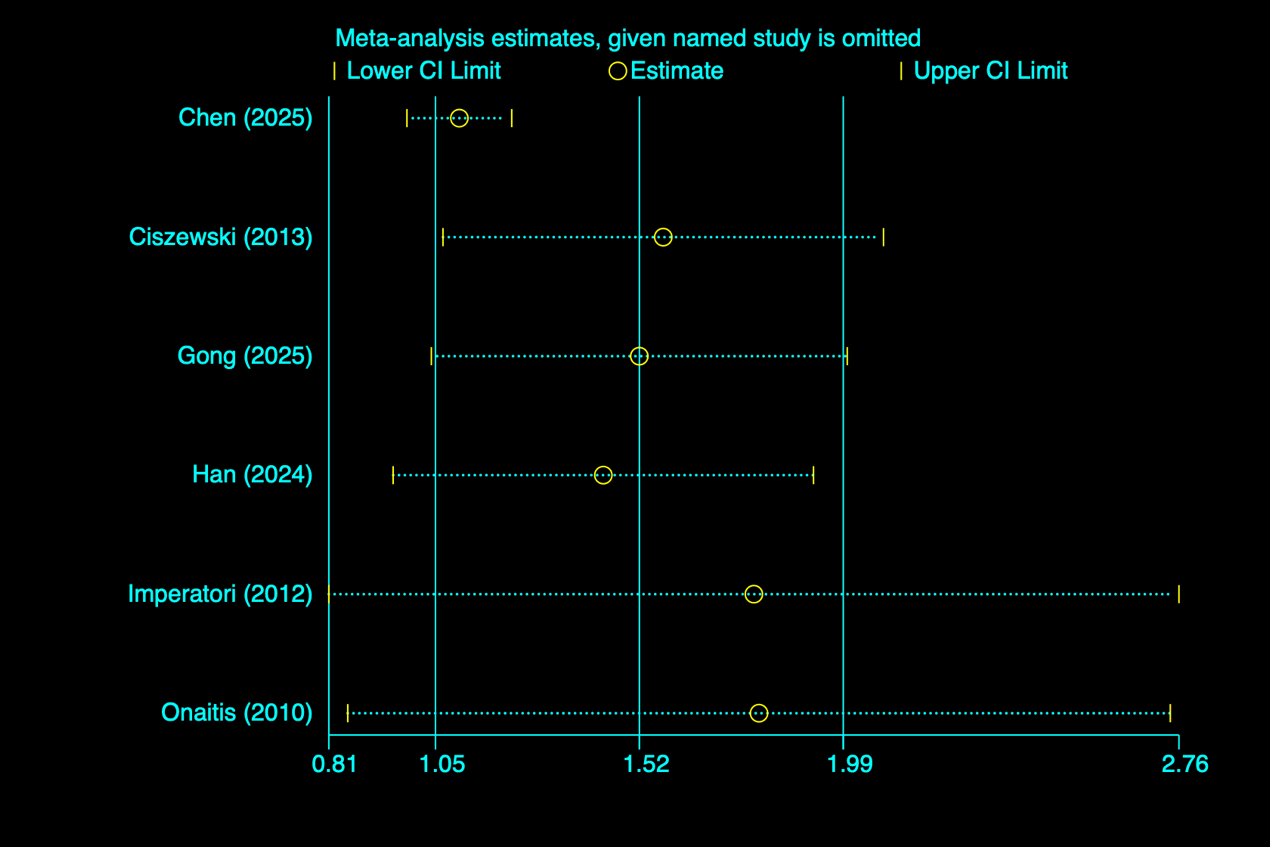


Figure S5 Sensitivity analysis for hypertension


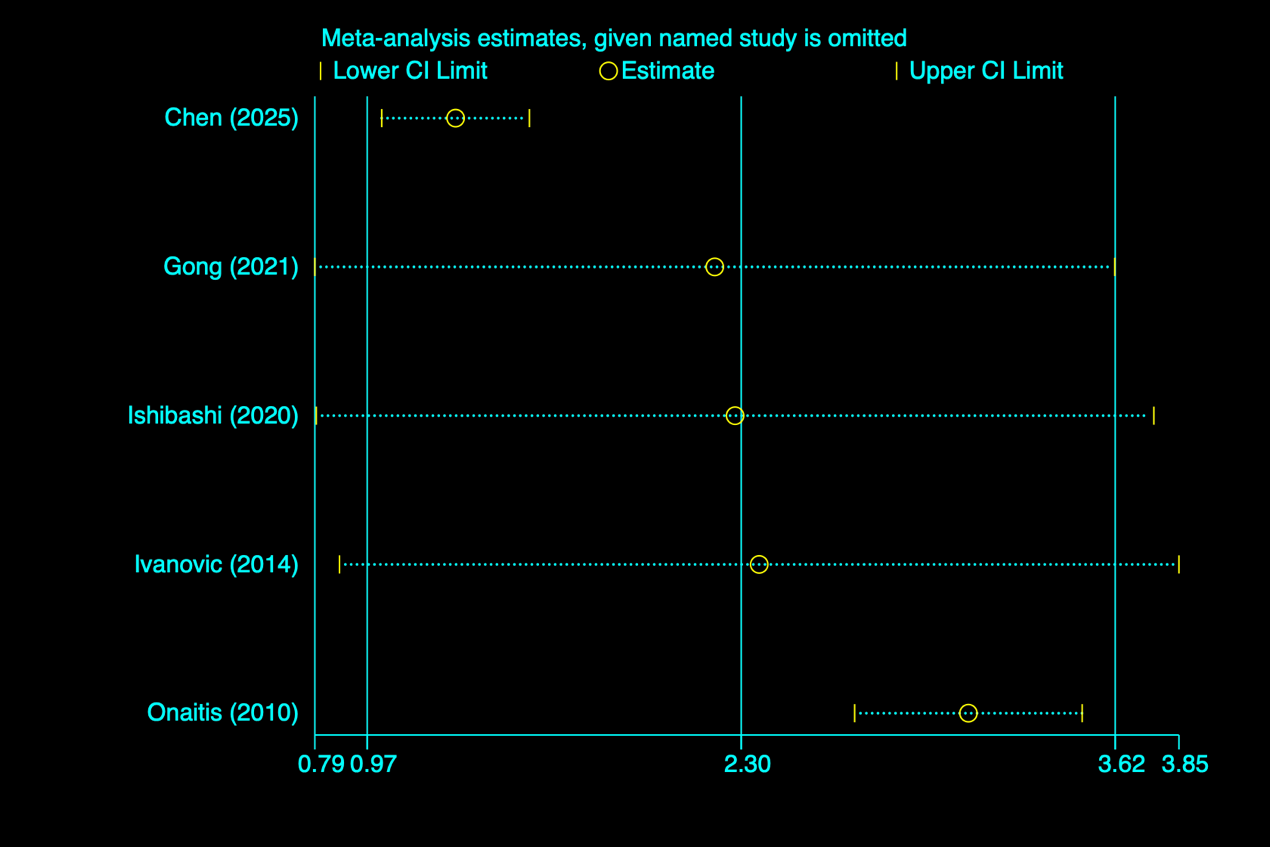


Figure S6 Sensitivity analysis for hypertension


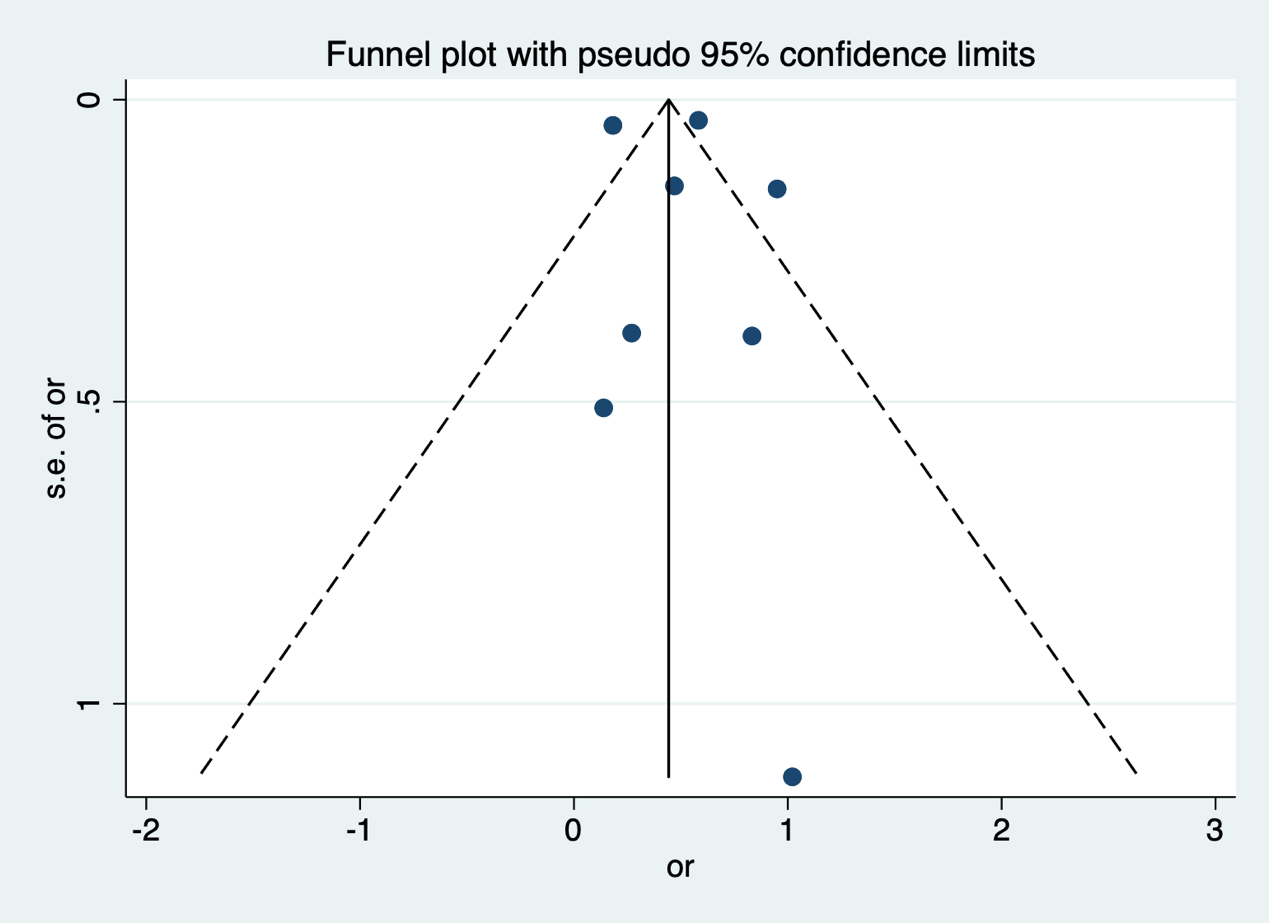


Figure S7 Funnel plot of meta-analysis of age>65


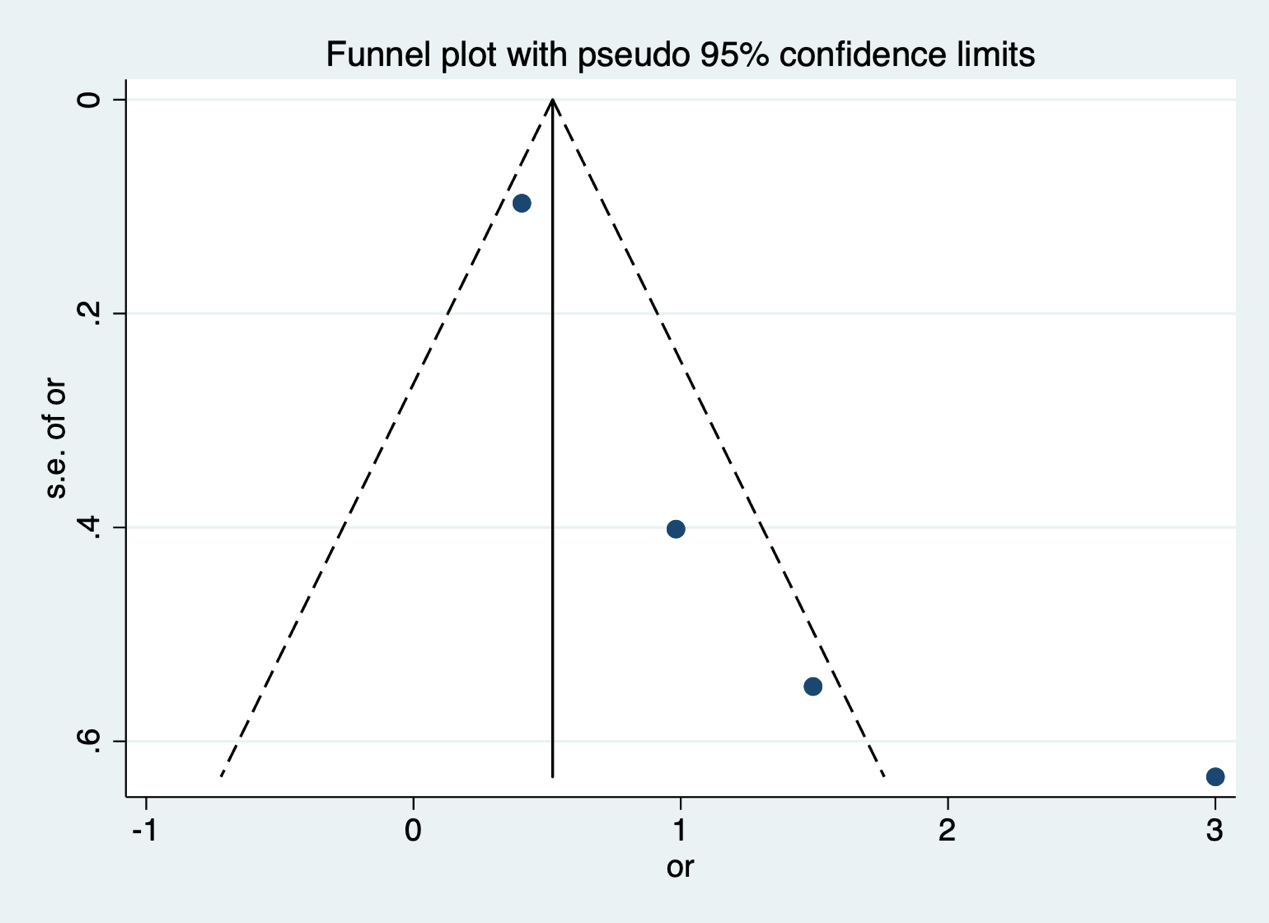


Figure S8 Funnel plot of meta-analysis of Postoperative high BNP


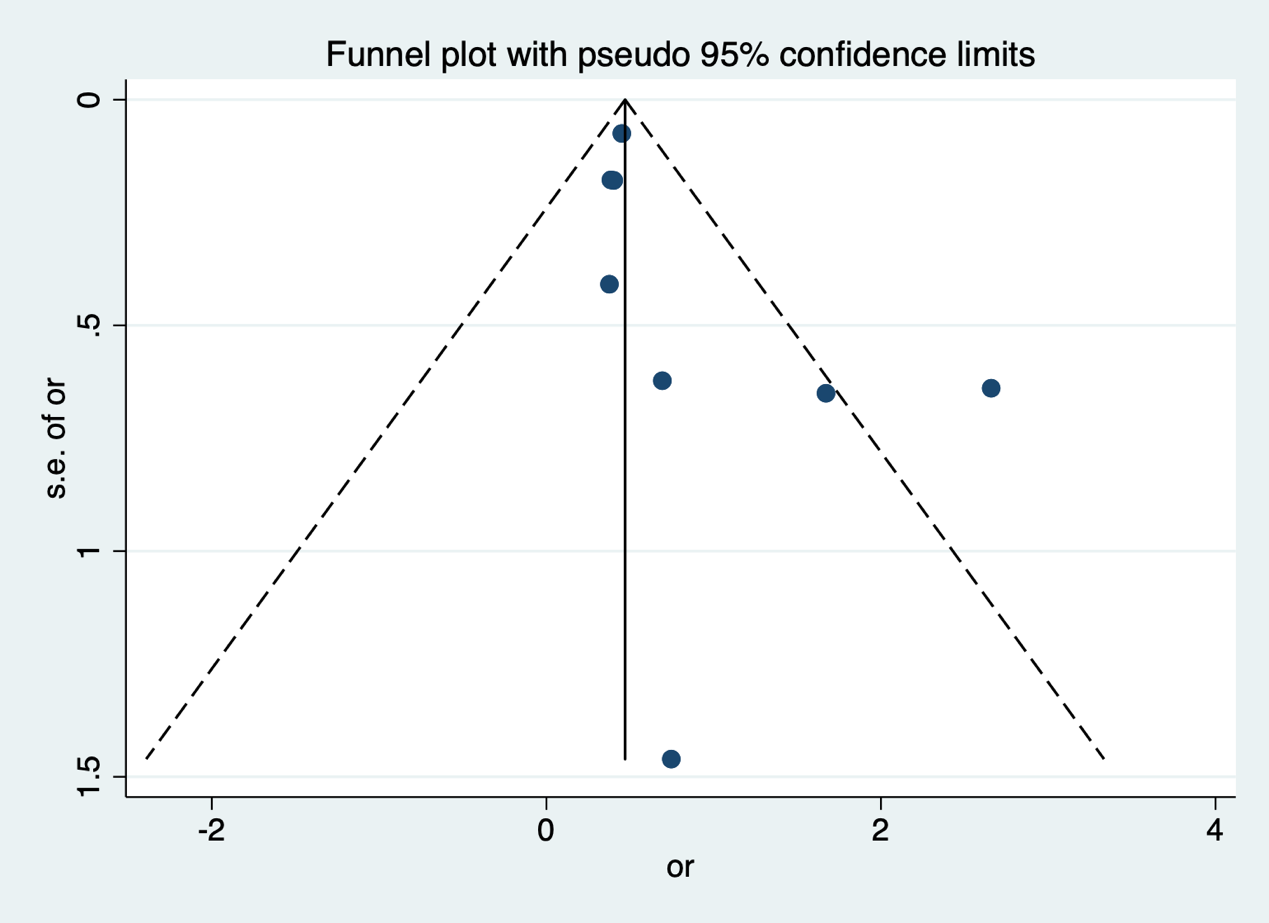


Figure S9 Funnel plot of meta-analysis of male


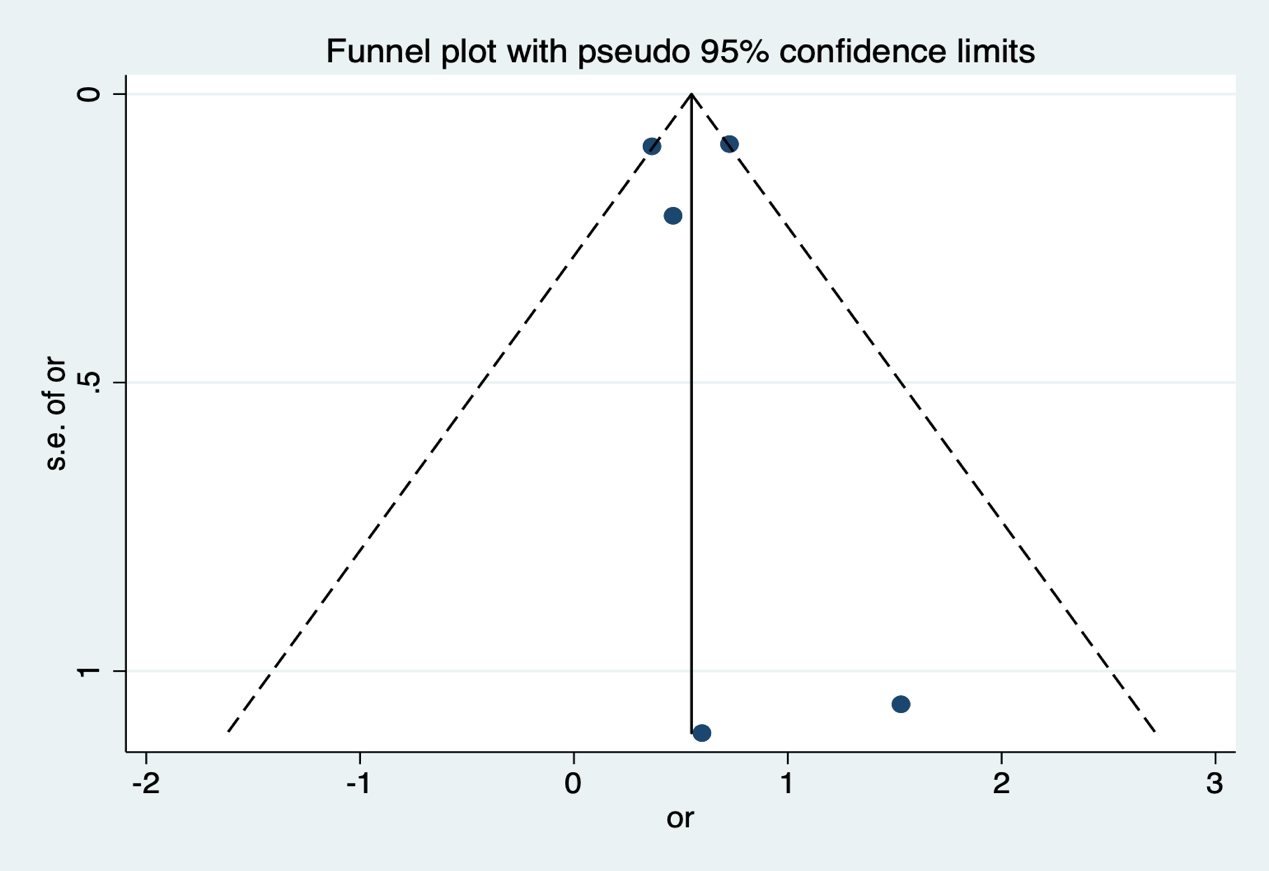


Figure S10 Funnel plot of meta-analysis of smoking


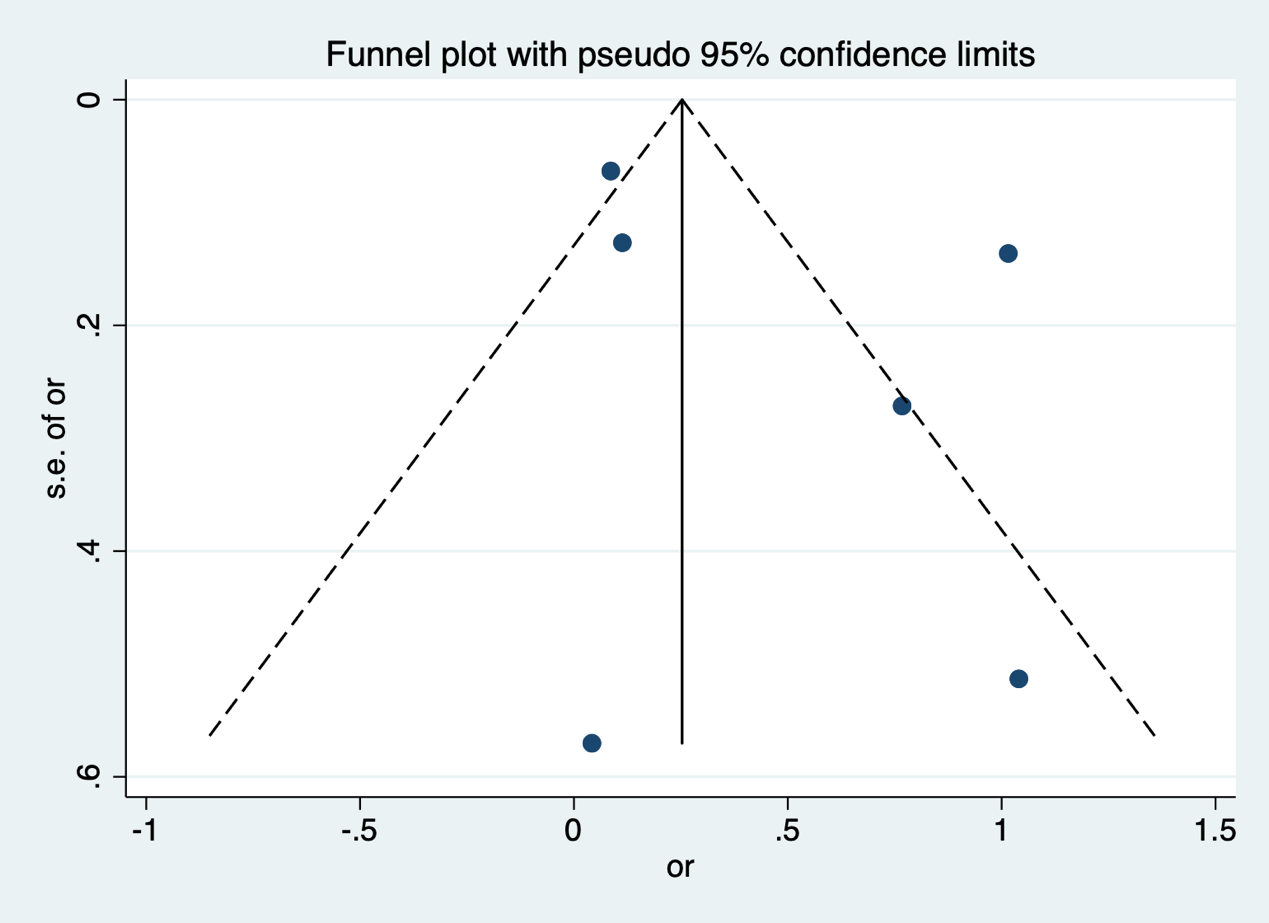


Figure S11 Funnel plot of meta-analysis of hypertension


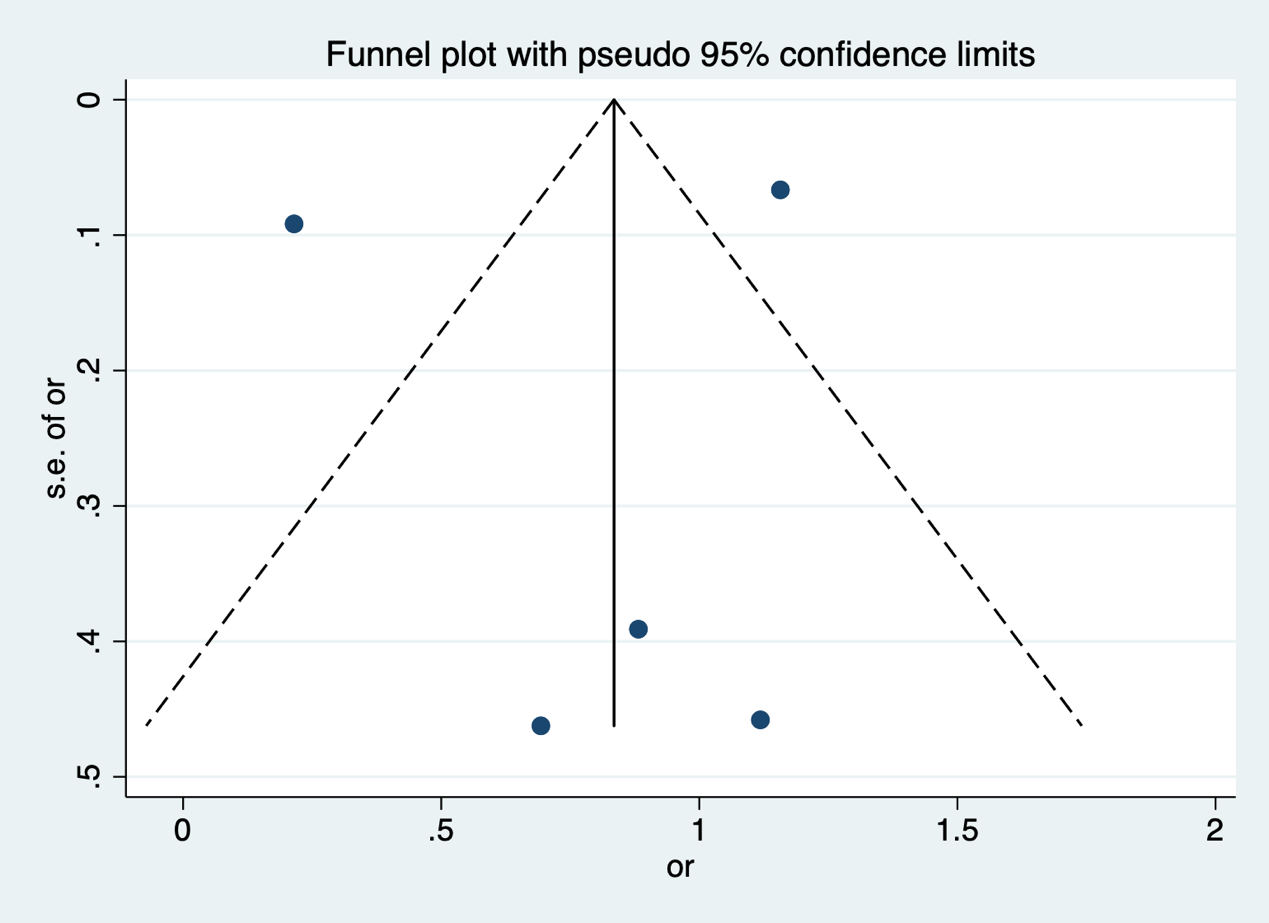


Figure S12 Funnel plot of meta-analysis of patients with TNM stage II lung cancer


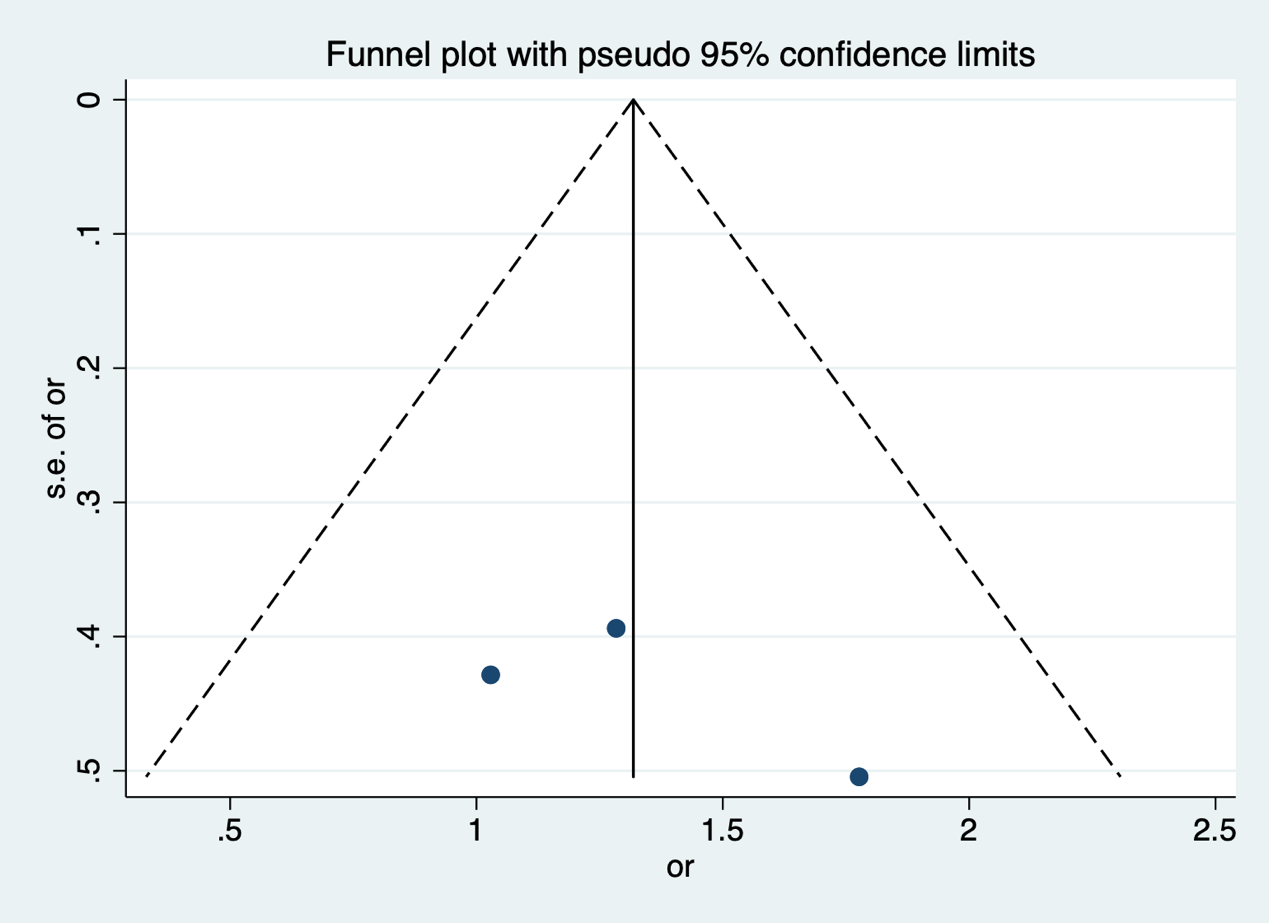


Figure S13 Funnel plot of meta-analysis of transfusion

Table S1 Meta-regression results

| Outcomes | Year | Country | study design |
| --- | --- | --- | --- |
| Age >65 years | 0.76 | 0.09 | 0.65 |
| Postoperative high BNP | 0.11 | 0.19 | 0.83 |
| hypertension | 0.38 | 0.82 | 0.28 |
| TNM stage II lung cancer | 0.15 | 0.66 | 0.07 |
